# Genomic Characterization of an Emerging SARS-CoV-2 Variant During the Early Second Wave of the SARS-CoV-2 Pandemic in Maharashtra, India

**DOI:** 10.7759/cureus.48604

**Published:** 2023-11-10

**Authors:** Rajesh P Karyakarte, Rashmita Das, Suvarna Joshi, Athira Jayaram, Sushma Yanamandra, Smriti Shende, Nyabom Taji, Srushti Rane, Reshma Bawale, Geetanjali P Chaudhari, Bhagyashree Karekar, Shivani R Sakalkar, Rahul G Tiwari, Madhuri G Jadhav

**Affiliations:** 1 Microbiology, B. J. Government Medical College & Sassoon General Hospitals, Pune, IND

**Keywords:** sars-cov-2 (severe acute respiratory syndrome coronavirus-2), n440k, p681r, l452r, e484q, sars-cov-2 whole-genome sequencing, kappa variant, b.1.617.1, clade 21b, covid-19

## Abstract

Background

The severe acute respiratory syndrome coronavirus-2 (SARS-CoV-2) has led to a global health crisis, with various variants emerging over time. In India, particularly in Maharashtra, a resurgence of cases and distinct transmission patterns have been observed. This study aimed to identify and characterize the circulating SARS-CoV-2 variants during the early second wave in Maharashtra, India.

Materials and methods

Nasopharyngeal swabs were collected from 24 RT-PCR-positive coronavirus disease of 2019 (COVID-19) cases across four districts of Maharashtra. Whole genome sequencing (WGS) was performed using the ARTIC amplicon sequencing protocol, and the data were analyzed.

Results

A total of 189 amino-acid mutations were identified across the 24 samples. Compared to the Indian genomes, 44 amino-acid mutations were unique to 24 genomes. Clade 20A was the most prevalent (66.66%), followed by 20B and 21B. The lineage B.1.36 (45.83%) was the most common, followed by B.1.617.1 (16.67%). The D614G mutation was the most frequent spike mutation (95.83%). Four samples from the Amravati district clustered distinctly under Clade 21B with spike mutations E154K in the N-terminal domain (NTD), L452R and E484Q in the receptor-binding domain (RBD) and P681R in proximity to the furin cleavage site. The temporal distribution of samples revealed the presence of Clade 21B in Maharashtra since the 31st of January 2021.

Conclusion

The study provides valuable insights into the circulating SARS-CoV-2 variants during the early second wave in Maharashtra, highlighting specific clades and mutations. The unique clustering patterns and the high prevalence of immune-escape mutations emphasize the need for continuous monitoring and genomic surveillance.

## Introduction

The severe acute respiratory syndrome coronavirus-2 (SARS-CoV-2), a novel beta-coronavirus causing COVID-19, has rapidly evolved into one of the most devastating health crises in recent history. It was in late December 2019 when the human cases of SARS-CoV-2 infection were identified in a group of patients with pneumonia of unknown origin. These cases were epidemiologically linked to the seafood market in Wuhan City, Hubei Province, China. The disease spread rapidly from Wuhan to other Chinese cities and other countries [1]. As a response, the World Health Organization (WHO) declared COVID-19 as a public health emergency of international concern (PHEIC) on the 30th of January 2020 [2]. On the same day, India reported its first case of COVID-19 in Kerala, with a travel history to Wuhan, China [3].

Since the beginning of the pandemic, the world has witnessed the emergence of various variants of concerns and interests. These have dominated different geographical areas in varying proportions, reflecting the natural evolution of the virus. This trend was also observed in India [4,5]. While the early months of the pandemic (March and April 2020) were dominated by Clade I/A3i, in the latter half of April 2020, the country saw a shift in clade prevalence, with most states showing an increased representation of Clade A2a [6]. The emergence of these variants could potentially become worrisome, as they might result in enhanced transmissibility, infectivity, or virulence [7].

During the first wave, the SARS-CoV-2 cases peaked in September 2020, with a steady decline in cases by the end of the year. However, in early January 2021, the country saw a resurgence in three states, namely, Maharashtra, Punjab, and Chhattisgarh. The seven-day trailing average national effective reproduction number, Rt, crossed the threshold of one on the 19th of February 2021. Around the same time, in late 2020, new SARS-CoV-2 variants were identified globally, notably the Alpha/B.1.1.7 variant in the United Kingdom, the Gamma/P.1 variant in Brazil, and the Beta/B.1.1351 variant in South Africa [8]. These new variants had raised concern, as they were feared to be driving the fresh surge in the abovementioned three Indian states. In Maharashtra, apart from the major cities such as Mumbai and Pune, districts in the Vidarbha region, notably Amravati, Nagpur, Yavatmal, and Buldhana, were reporting an increase in the number of COVID-19 cases and deaths. The situation was perplexing in the Amaravati district as entire families were getting infected, unlike during the first wave. Unlike cities such as Mumbai and Pune, the district does not have a high population density and is far less crowded, with a limited inflow of people from other regions. Despite these factors, the positivity rate in the district had soared to 50% [9].

Therefore, considering the worldwide emergence of SARS-CoV-2 variants and the surge in the number of cases in Maharashtra, the current study was undertaken to identify and characterize the circulating SARS-CoV-2 variants that were contributing to the increase in cases in the state.

## Materials and methods

Sample collection and viral RNA extraction

Nasopharyngeal swabs were collected from 24 reverse transcriptase polymerase chain reaction (RT-PCR)-positive COVID-19 cases, with a cycle threshold (Ct) value less than 25, across four districts of Maharashtra, India. The samples were selected from the districts of Amravati (four samples), Yavatmal (four samples), Satara (four samples), and Pune (12 samples). The collection dates for these samples ranged from the 23rd of December 2020 to the 7th of February 2021. The criteria for the selection of samples were based on the spread, severity, and incidence of COVID-19 infection in the respective districts. A specimen referral form (SRF) was used to collect all relevant metadata, which was filled in at the time of collection of nasopharyngeal swabs.

The samples were collected in a viral transport medium (VTM). They were transported to the molecular laboratory at Byramjee Jeejeebhoy Government Medical College, Pune, maintaining a cold chain at 2-8 °C and adhering to the standard operating procedures (SOP) prescribed by the Indian Council of Medical Research [10].

Viral RNA was extracted using MagRNA-II Viral RNA Extraction Kit (Genes2Me Private Limited, Gurgaon, Haryana, India) following the manufacturer’s instructions. The extracted RNA was transported in a cold chain to the molecular laboratory at Genotypic Technology Private Limited, Bangalore, India, where SARS-CoV-2 whole genome sequencing (WGS) was performed.

Library preparation and next-generation sequencing

The SARS-CoV-2 whole genome sequencing protocol was based on the ARTIC amplicon sequencing protocol developed by Josh Quick [11]. In brief, tiled PCR amplicons were generated from cDNA with the primers designed using the PrimalScheme (https://primalscheme.com/). The SARS-CoV-2 library was prepared using the Native Barcoding kit (EXP-NBD114; Oxford Nanopore Technologies, Oxford, England) and Ligation kit (SQK-LSK109) (Oxford Nanopore Technologies, Oxford, England). The cDNA was end-repaired using NEBNext Ultra II End repair/ dA-tailing Module (NEB#E7546L; New England Biolabs, Ipswich, MA), followed by purification with 0.4X AMPure XP beads. The samples were barcoded using the NEBNext Ultra II Ligation Module (NEB# E7595; New England Biolabs, Ipswich, MA) and Native Barcoding Kit (EXP-NBD104). The barcoded samples were purified using 0.4X AMPure-XP beads. Further, sequencing adapter ligation was performed using the NEBNext Quick Ligation Module (NEB# E6056L; New England Biolabs, Ipswich, MA), followed by purification using 0.4X AMPure-XP beads. The final library was eluted in 15 μL of elution buffer and was loaded on the SpotON flow cell, FLO-MIN106, for sequencing.

Sequencing was performed on GridION X5 (Oxford Nanopore Technologies, Oxford, England) following a 48-hour sequencing protocol on GridION Release 19.06.9 (Oxford Nanopore Technologies, Oxford, England). The raw nanopore reads (‘fast5’ format) were base called (‘fastq’ format) and de-multiplexed using GUPPY v3.2.2 (Oxford Nanopore Technologies, Oxford, England). The raw data generated were subjected to analysis using the ARTIC protocol.

Genomic data analysis and visualization

Variant Calling, Annotation, and Lineage Analysis

The base-called reads were analyzed using the ARTIC protocol [11] to generate consensus SARS-CoV-2 genomes. In brief, the processed data were mapped against the SARS-CoV-2 reference genome (Wuhan Strain Accession ID: MN908947.3) using the Minimap2 tool (https://github.com/lh3/minimap2). The aligned data were further processed using SAMtools (www.htslib.org) to generate consensus sequences. Nanopolish was used for variant calling, and the identified variants were annotated using the SNPEff tool (https://bio.tools › snpeff). The generated consensus sequences were subjected to clade and lineage analysis using the Nextclade software (version 2.3.0; Microbial Evolution group, Wageningen, Netherlands) and Pangolin COVID-19 Lineage Assigner (version 4.1.1; https://pangolin.cog-uk.io/) and pangolin-data (version 1.12), respectively.

Genomic Data Collection and Phylogenetic Analysis

The datasets of Indian SARS-CoV-2 genomes deposited in the Global Initiative on Sharing All Influenza Data (GISAID) database until the 28th of February 2021 were utilized for the present study [12]. Only high-quality sequences with complete genome coverage and metadata were retrieved from the database for further analysis ((Appendix 1; accessible at: doi.org/10.55876/gis8.230918nw).

Phylogenetic analysis of the 24 samples and the sequences retrieved from the GISAID database was performed according to the Nextstrain pipeline. The sequences were clustered and aligned against the reference genome MN908947.3 using Augur, the phylodynamic pipeline provided by Nextstrain. The initial phylogenetic tree was constructed using IQTREE 2 (version v2.2.2.6; http://www.iqtree.org/) following the Augur tree framework provided by Nextstrain [13]. The resulting tree was visualized and annotated using iToL (https://itol.embl.de), a web-based tool commonly used for interactive tree visualization and annotation [14].

## Results

Demographic and clinical characteristics of the samples sequenced

Table 1 describes the demographic characteristics of the 24 samples included in the study. The participants had a mean age of 38.92 ± 16.99 years, with a male-to-female ratio of 1:1.18. The sample collection dates for the 24 samples ranged from the 23rd of December 2020 to the 7th of February 2021. Out of the 24 cases, 12 (50%) cases were found to have an asymptomatic infection, while the remaining 12 (50%) cases had symptomatic disease. Among those with symptoms, three cases (25%) were diagnosed with severe acute respiratory illness (SARI), and the remaining nine cases (75%) presented with influenza-like illness (ILI). The primary mode of infection was through close contact with infected individuals indicating the community spread. Of the 24 cases, 23 individuals (95.83%) survived, and one (4.17%) died. The individual who died was a five-year-old female with underlying heart disease and was diagnosed with SARI.

**Table 1:** Demographic and clinical characteristics of the 24 individuals

Demographic characteristics	Total Count (%)
1. Age (in years)	
0-9	2 (8.33%)
10-19	2 (8.33%)
20-29	2 (8.33%)
30-39	5 (20.83%)
40-49	5 (20.83%)
50-59	5 (20.83%)
60-69	3 (12.50%)
2. Gender	
Male	11 (45.83%)
Female	13 (54.17%)
3. Date of sample collection	
Week 52, 2020 (21^st^ December 2020 to 27^th^ December 2020)	1 (4.17%)
Week 04, 2021 (25^th^ January 2021 to 31^st^ January 2021)	7 (29.17%)
Week 05, 2021 (1^st^ February 2021 to 7^th^ February 2021)	16 (66.66%)
4. Area of Residence	
Amravati	4 (16.67%)
Yavatmal	4 (16.67%)
Satara	4 (16.67%)
Pune	12 (50%)
Clinical Characteristics	
1. Symptom status at the time of sample collection	
Asymptomatic	12 (50%)
Symptomatic	12 (50%)
Severe Acute Respiratory Illness (SARI)	3 (25%)
Influenza-like Illness (ILI)	9 (75%)
2. Outcome of disease	
Survived	23 (95.83%)
Dead	24 (4.17%)

Sequencing and data processing

A total of 24 samples were processed for SARS-CoV-2 whole genome sequencing. The amplicon-based sequencing protocol generated an average of 0.32 million reads per sample, with an average median read length of 519.48 base pairs. The reads had an average genome coverage of 93.63%, with a minimum read depth of 10 (10X) and an average depth of 5397.57X per sample.

Variant annotation and classification of variants

**Figure 1:**
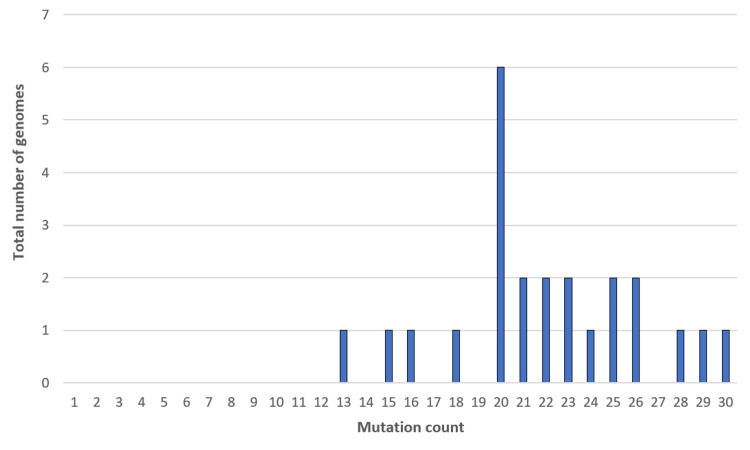
Distribution of mutation counts across the 24 SARS-CoV-2 genomes

**Figure 2:**
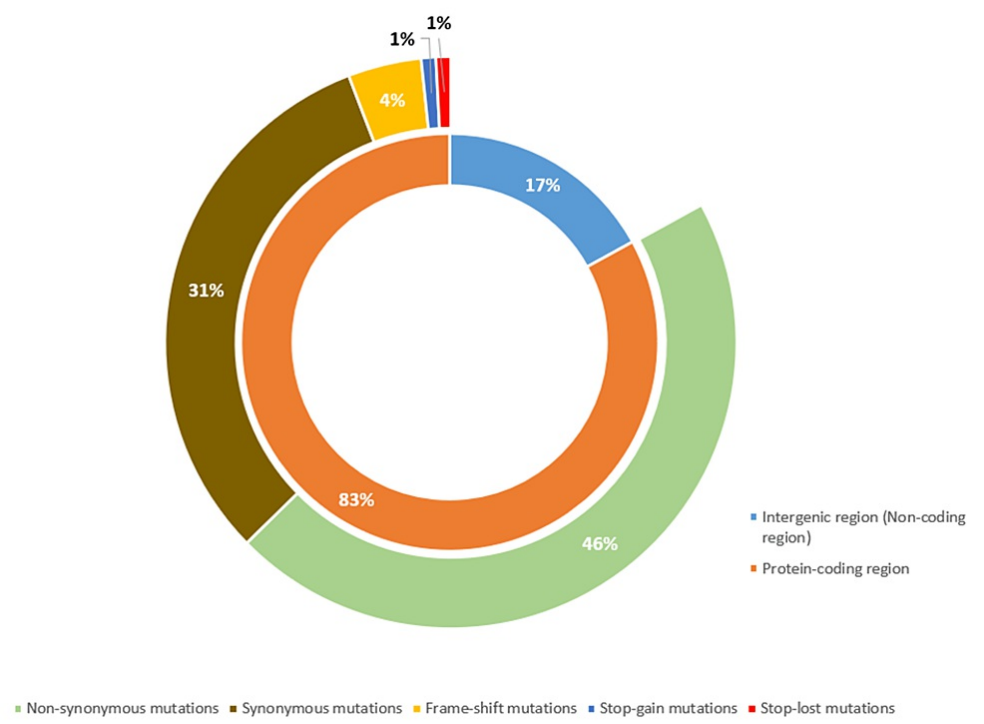
Classification of unique mutations identified in 24 SARS-CoV-2 genomes

The 24 sequenced samples were further processed to detect variants and generate consensus sequences. Upon analysis, 189 mutations were identified in 24 samples, with a median mutation count of 21.5 per sample. Figure 1 illustrates the distribution of the mutation counts across the 24 sequenced genomes. Out of the 189 mutations identified, 156 (82.54%) were in the protein-coding regions of the genome, while the remaining 33 (17.46%) mutations were present in the intergenic regions (non-coding regions). Within the protein-coding regions, 86 (55.13%) were identified as non-synonymous mutations, altering the amino-acid sequence. Further, 59 (37.82%) were synonymous mutations, seven (4.49%) were frame-shift mutations, and two (1.28%) mutations each were stop-gain and stop-loss mutations (Figure 2). Of the 156 protein-coding mutations, 86 (55.13%) were located in the ORF1ab gene, 30 (19.23%) in the spike (S) gene, and the remaining 40 (25.64%) mutations were found in other genes such as the nucleocapsid (N), membrane (M), ORF3a, ORF6, ORF7a, and ORF8 genes.

Clade and lineage analysis of 24 samples, along with Indian SARS-CoV-2 genomes submitted on GISAID as of 28 February 2021

**Figure 3:**
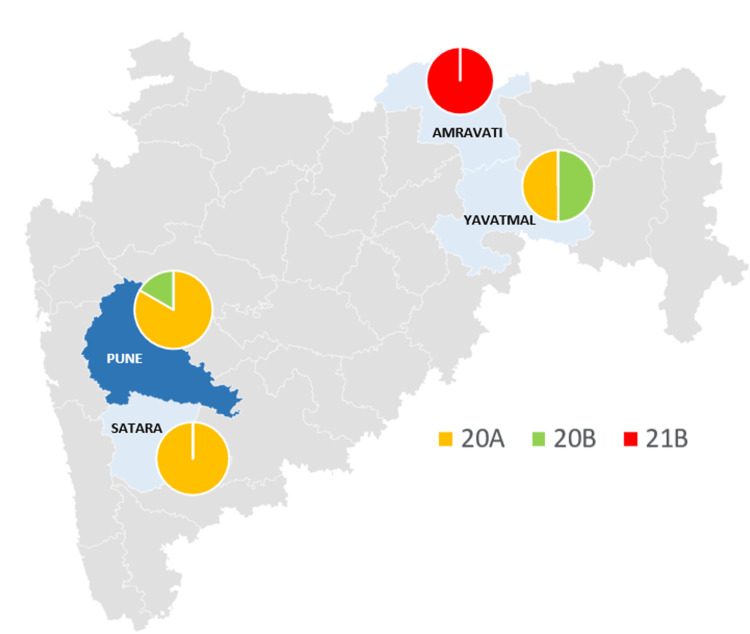
Distribution of different clades among 24 samples sequenced from Maharashtra

Among the 24 SARS-CoV-2 samples, as per the current clade designation, Clade 20A emerged as the most prevalent clade (66.66%), followed by 20B and 21B, each making up 16.67% of the samples. Similarly, in terms of the current Pangolin lineage, B.1.36 (45.83%) was the most common lineage found, followed by B.1.617.1 (16.67%), B.1.1.216 (12.5%), and B.1.36.29 (12.5%). Table 2 describes the distribution of these clades and lineages across different districts. Specifically, every sample (100%) from the Amravati district belonged to lineage B.1.617.1 (Clade 21B), the WHO variant Kappa. In Yavatmal, the samples were evenly distributed between lineage B.1.36.22 (Clade 20A) and B.1.1.216 (Clade 20B), each constituting 50% of the total. Contrarily, lineage B.1.36 (Clade 20A) was the dominant lineage in samples from Satara (100%) and Pune (70%) (Figure 3).

**Table 2:** District-wise distribution of Nextstrain clades and Pangolin lineages in 24 SARS-CoV-2 samples

Districts of Maharashtra	Nextclade Clade		Pangolin Lineage		Total Count of Variant (%)	Total Count of Samples (%)	
	Designation at the time of study	Current designation	Designation at the time of study	Current designation			
Amravati	20A	21B	B.1	B.1.617.1	4 (100%)	4 (16.66%)	
Yavatmal	20A	20A	B.1.36	B.1.36.22	2 (50%)	4 (16.66%)	
	20B	20B	B.1.1.216	B.1.1.216	2 (50%)		
Satara	20A	20A	B.1.36	B.1.36	4 (100%)	4 (16.66%)	
Pune	20A	20A	B.1.36	B.1.36	7 (70%)	10 (83.33%)	12 (50%)
				B.1.36.22	2 (20%)		
				B.1.36.29	1 (10%)		
	20B	20B	B.1.1.216	B.1.1.216	1 (50%)	2 (16.67%)	
			B.1.1.306	B.1.1.306	1 (50%)		

**Figure 4:**
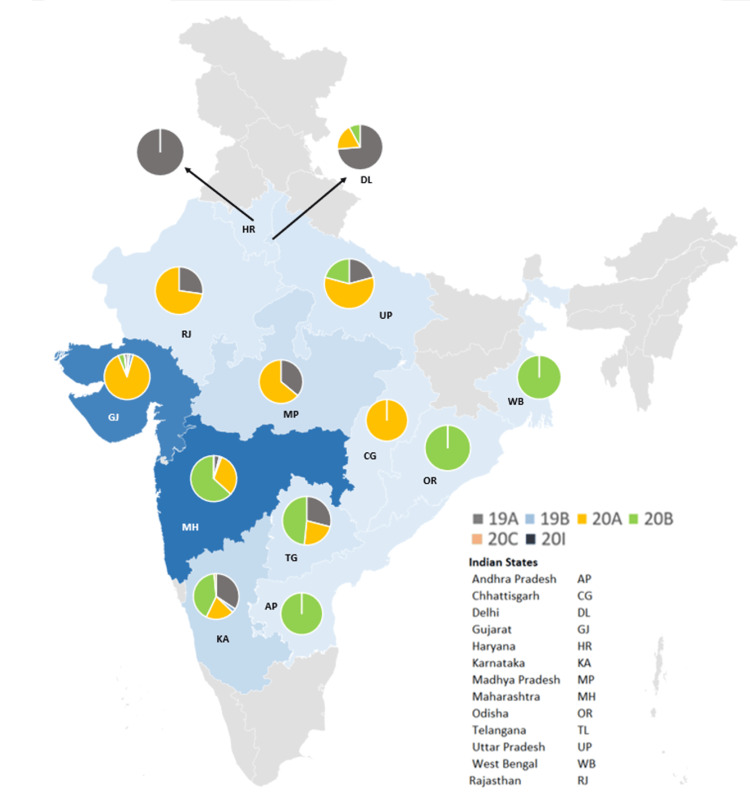
Distribution of different clades among 1,474 Indian SARS-CoV-2 sequences downloaded from GISAID (as of 28 February 2021)

A total of 1,474 sequences, submitted on GISAID as of 28 February 2021, were downloaded from the database. On analyzing the 1,474 sequences, 673 (45.66%) sequences were from Maharashtra, 588 (39.89%) from Gujarat, and 213 (14.45%) sequences were from other states within the country. Clade 20A was the dominant clade (54.34%), followed by 20B (34.40%), 19A (8.48%) and 19B (1.90%). In the regional breakdown, while Clade 20A was more prevalent in Gujarat, Madhya Pradesh, Rajasthan, Chhattisgarh, and Uttar Pradesh, sequences from Maharashtra, Andhra Pradesh, Odisha, and West Bengal were dominated by Clade 20B. Conversely, Clade 19A was predominantly seen in Haryana and Delhi (Figure 4). With regard to Pangolin lineages, B.1.1.306 (27.61%) was the most prevalent lineage, followed by B.1 (16.76%), B.1.36.8 (14.59%), and B.1.210 (13.64%). It is important to note that, among the sequences uploaded on GISAID as of 28 February 2021, Clade 21B was not detected. However, it was identified for the first time in India in samples sequenced in the present study. These samples, identified as Clade 21B, were collected from suspected SARI/influenza-like illness (ILI)-hospitalized patients. These patients were from different parts of the Amravati district, emphasizing the community spread of the novel variant within the region. Table 3 describes the clade-wise distribution of age, gender, and clinical outcome of 1,474 cases.

**Table 3:** Clade-wise distribution of age, gender, and clinical outcome of 1,474 cases

	19A	19B	20A	20B	20C	20I	Grand Total
1. Age-wise distribution (in years)							
0-9	02 (1.6%)	01 (3.57%)	17 (2.12%)	16 (3.16%)	0 (0%)	1 (1.11%)	37 (2.51%)
10-19	12 (9.6%)	03 (10.71%)	31 (3.87%)	30 (5.92%)	0 (0%)	0 (0%)	76 (5.16%)
20-29	34 (27.2%)	04 (14.29%)	99 (12.36%)	91 (17.95%)	0 (0%)	0 (0%)	228 (15.47%)
30-39	19 (15.2%)	03 (10.71%)	155 (19.35%)	103 (20.32%)	1 (25%)	3 (33.3%)	284 (19.27%)
40-49	14 (11.2%)	04 (14.29%)	162 (20.22%)	77 (15.19%)	1 (25%)	3 (33.3%)	261 (17.71%)
50-59	19 (15.2%)	04 (14.29%)	161 (20.10%)	98 (19.33%)	0 (0%)	0 (0%)	282 (19.13%)
60-69	19 (15.2%)	08 (28.57%)	110 (13.73%)	59 (11.64%)	2 (50%)	0 (0%)	198 (13.43%)
70-79	05 (4%)	01 (3.57%)	55 (6.87%)	21 (4.14%)	0 (0%)	2 (22.2%)	84 (5.70%)
> 80	01 (0.8%)	0 (0%)	11 (1.37%)	12 (2.37%)	0 (0%)	0 (0%)	24 (1.63%)
2. Gender-wise distribution							
Female	43 (34.4%)	10 (35.71%)	251 (31.34%)	197 (38.86%)	2 (50%)	4 (44.4%)	507 (34.40%)
Male	82 (65.6%)	18 (64.29%)	550 (68.66%)	310 (61.14%)	2 (50%)	5 (55.6%)	967 (65.60%)
3. Clinical outcome of disease							
Alive	121 (96.80%)	27 (96.43%)	730 (91.14%)	487 (96.06%)	3 (75%)	9 (100%)	1377 (93.42%)
Dead	4 (3.20%)	1 (3.57%)	71 (8.86%)	20 (3.94%)	1 (25%)	0 (0%)	97 (6.58%)

Phylogenetic analysis of 24 samples and Indian SARS-CoV-2 genomes submitted on GISAID as of 28 February 2021

**Figure 5:**
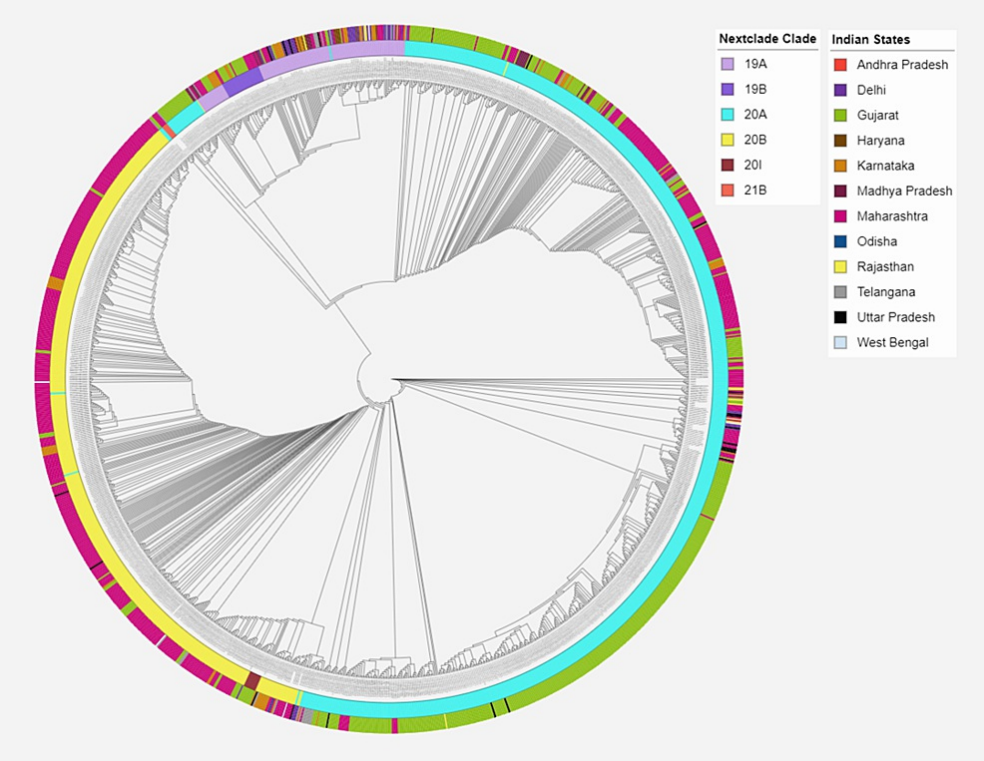
Phylogenetic relationship between the Indian genomes and the 24 genomes sequenced in the study

Further, to understand the evolutionary relationships among the variants, 24 samples were analyzed along with 1,474 high-coverage Indian SARS-CoV-2 whole genome sequences with complete genome coverage and metadata submitted on the GISAID database as of 28 February 2021. Among the genomes sequenced in the present study, 16 genomes (two from Yavatmal, four from Satara, and 10 from Pune) were found to cluster under the Nextstrain Clade 20A, the dominant clade in the Indian sequences. Four genomes (two from Yavatmal and two from Pune) clustered under Clade 20B. Notably, the four samples from the Amravati district were distinctly clustered under Clade 21B. The phylogenetic relationship between the Indian genomes and the 24 genomes sequenced in the study is summarized in Figure 5.

Temporal distribution of SARS-CoV-2 clades during the study period (Indian SARS-CoV-2 genomes submitted on GISAID as of 28 February 2021)

**Figure 6:**
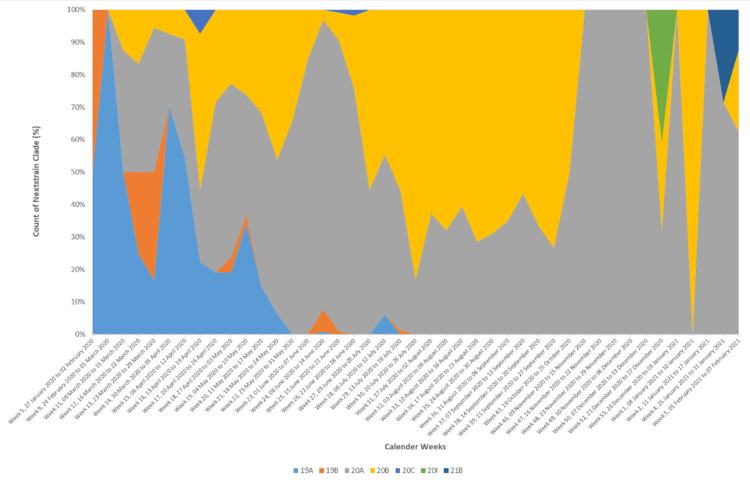
Temporal distribution of SARS-CoV-2 clades in India

To understand the temporal distribution of SARS-CoV-2 clades, the cumulative counts of the clades were plotted against the sample collection date. By the end of May 2020, Clades 19A and 19B were entirely replaced by Clades 20A and 20B, dominating the remaining part of the wave. The samples belonging to Clade 21B, which were identified for the first time during the present study, were collected on 31 January 2021 and 2 February 2021 in Maharashtra (Figure 6).

Mutation analysis of 24 samples sequenced during the study

**Figure 7:**
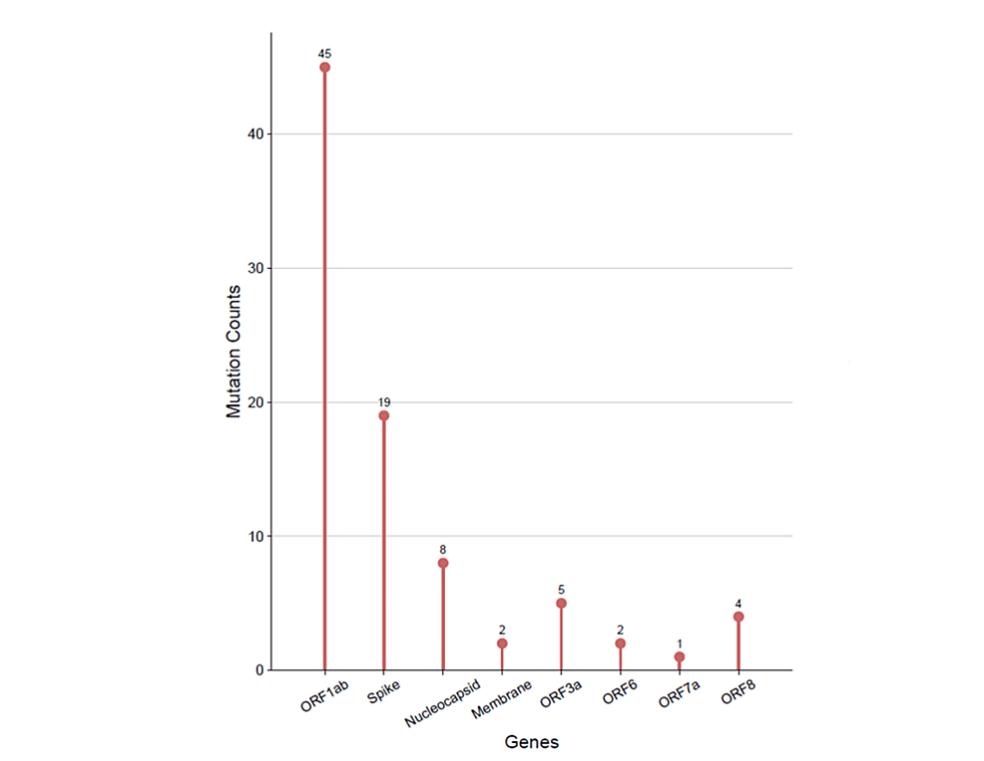
Distribution of non-synonymous mutations identified in 24 SARS-CoV-2 genomes

**Figure 8:**
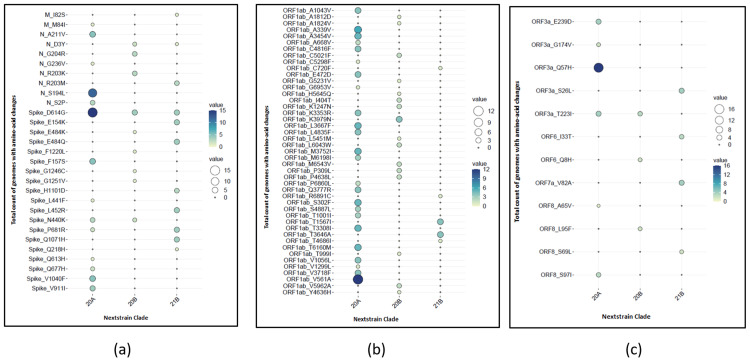
Clade-wise prevalence of amino acid changes across the 24 SARS-CoV-2 genomes (a) Amino acid changes in the structural genes (M, N, and S genes). (b) Amino acid changes in the non-structural genes (ORF1a and ORF1b). (c) Amino acid changes in the accessory genes (ORF3a, ORF6, ORF7a, and ORF8)

A total of 86 non-synonymous mutations were identified in 24 SARS-CoV-2 genomes. Among these, 45 were found in the non-structural genes (ORF1ab), 29 were in structural genes (including spike, membrane, and nucleocapsid genes), and the remaining 12 were found in the accessory genes (Figure 7). The clade-wise prevalence of amino acid changes across the 24 SARS-CoV-2 genomes is summarized in Figure 8. Notably, the D614G mutation was the most frequent spike mutation (95.83%) observed across all the clades. In the receptor-binding domain (RBD), five distinct mutations were identified. Specifically, mutations E484Q and L452R were present in all the samples (100%) belonging to Clade 21B. Mutation N440K was identified in 50% of samples of Clade 20B and 18.75% of samples of Clade 20A. Other identified RBD mutations included E484K in 25% of samples of Clade 20B and L441F in 6.25% of samples from Clade 20A. The P681R mutation, a mutation close to the furin cleavage site, was identified in all the samples (100%) of Clade 21B and in 12.5% of samples of Clade 20A. Similarly, the mutations in the ORF1ab region revealed distinct characteristics for each clade. Clade 20A had the highest number of ORF1ab mutations (24), followed by Clade 20B (16) and Clade 21B (five). This distribution underscores the variability in the ORF1ab mutations across different clades, thereby depicting the unique mutational patterns associated with each clade.

**Figure 9:**
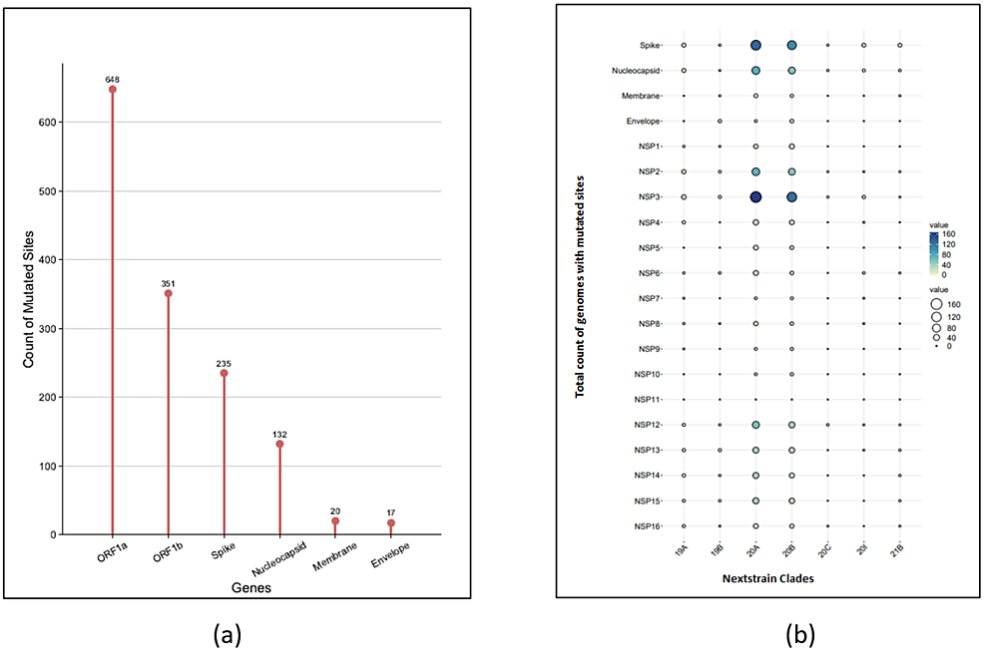
Total count of amino-acid mutations present in different regions of the SARS-CoV-2 genome (n=1,498) (a) Plot showing the total number of amino acid mutations identified in each region of the SARS-CoV-2 genome (number of genomes analyzed is 1,498). (b) Clade-wise distribution of amino acid mutations across different regions of the SARS-CoV-2 genome (number of genomes analyzed is 1,498)

On analyzing the 1,498 sequences (24 sequences from the current study and 1,474 sequences downloaded from the GISAID database), a total of 1,403 amino acid mutations were identified, of which ORF1a was the most frequently mutated region (46.19%), followed by ORF1b (25.02%), spike (16.75%), and nucleocapsid (9.41%) region in SARS-CoV-2 genomes from India (Figure 9a). Figure 9b presents the clade-wise distribution of mutated regions across the SARS-CoV-2 genome.

Of the 1,403 mutations identified, 784 (55.88%) were unique, occurring only in single sequences. While 11 (0.78%) mutations were found in 5% or more genomes, the remaining 608 (43.34%) mutations were rare and occurred in less than 5% of genomes submitted to GISAID. Compared to the Indian genomes, in the present study, we identified 44 mutations unique to the 24 samples. Among these mutations, 18 (40.91%) were identified exclusively in Clade 20A, 10 (22.73%) in Clade 20B, and 13 (29.54%) in Clade 21B. The remaining three (6.82%) mutations were seen in either of the clades. Notably, Clade 21B had unique mutations T95I, E154K, and G142D in the N-terminal domain (NTD), L452R in the receptor binding domain (RBD) of the spike protein, and P681R in the furin cleavage site. Other distinct spike mutations identified include Q1071H and H1101D.

Clade patterns versus clinical symptom analysis in 24 cases

On analysis of the symptoms of 24 cases with the SARS-CoV-2 clades, it was observed that 75% of asymptomatic cases were infected with Clade 20A. On the other hand, among the symptomatic cases, 58.33% of cases were infected with Clade 20A, and 33.33% were infected with Clade 21B. Further, among the symptomatic cases diagnosed as SARI, 66.67% had Clade 21B infection; among the ILI cases, 77.78% had Clade 20A infection (Table 4).

**Table 4:** Distribution of Nextstrain clades in asymptomatic and symptomatic SARS-CoV-2 cases

Nextstrain Clades	20A	20B	21B	Total
Asymptomatic disease	9 (56.25%)	3 (75%)	0	12 (50%)
Symptomatic disease	7 (43.75%)	1 (25%)	4 (100%)	12 (50%)
ILI	7 (100%)	0	2 (50%)	9 (75%)
SARI	0	1 (100%)	2 (50%)	3 (25%)
Total count	16 (100%)	4 (100%)	4 (100%)	24 (100%)

## Discussion

Like other RNA viruses, coronaviruses undergo rapid evolution, which can be observed and measured over months or years. This evolution occurs on timescales comparable to its transmission events and ecological dynamics. The primary driving force behind viral evolution is the rate at which the virus accumulates mutations and spreads through the population. For SARS-CoV-2, as with other beta coronaviruses, the estimated mutation rate ranges from 1 x 10^-6^ to 2 x 10^-6^ mutations per nucleotide per replication cycle. While many mutations are deleterious for viral replication, others are positively selected to persist in the population [15]. For instance, the D614G mutation in the spike protein, which quickly became the most widespread mutation of SARS-CoV-2, reportedly enhances viral shedding, viral fitness, and infectivity. Several mutations in the RBD region of the spike protein, such as N501Y (found in B.1.17 and B.1.351 lineages), N501T, and N501S, have been independently selected and are known to play a significant role in angiotensin-converting enzyme 2 (ACE2) binding, antibody recognition, enhanced transmission, and infectivity [7]. Additionally, K417N and E484K, found in B.1.351, contribute to the evasion of neutralization by multiple monoclonal antibodies [16]. The nsp1, also known as the leader protein, is central to inhibiting the antiviral innate immune response, so mutations in this region can impact viral pathogenicity [17]. Hence, it is essential to vigilantly track the evolution of the virus and understand the implications of these changes on viral transmission, pathogenesis, and vaccine and therapeutic efficacies.

At the time of the present study, what is today known as Clade 21B or lineage B.1.617.1 was initially classified as Clade 20A and lineage B.1 by Nextclade and Pangolin, respectively. The detection of unique mutations in four distinct samples from unrelated individuals in Amravati, along with a surge in local cases signaled the possibility of a new variant circulating in the community, which was communicated to the government immediately [18]. The variant was officially designated as lineage B.1.617.1 by Pangolin on 4 June 2021 [19] and Clade 21B on 8 June 2021 by Nextclade [20]. The unique mutations identified in the NTD and the RBD of the spike protein, specifically clustered in Clade 21B, lineage B.1.617.1. These included E154K in the NTD, L452R, and E484Q in the RBD and P681R in proximity to the furin cleavage site. Some of these mutations have been detected in other variants separately, such as L452R in B.1.427 (Epsilon) and B.1.429 variants and E484K (similar to E484Q) in the B.1.351 variant. However, the coexistence of L452R and E484Q in B.1.617.1 is unprecedented [16]. The RBD’s vital functional and antigenic properties make structural changes in this domain particularly significant. These mutations in the RBD have demonstrated increased binding affinity to ACE2 and disruption of interaction with neutralizing antibodies, leading to enhanced transmissibility and immune evasion. The P681R mutation at the furin cleavage site is known to enhance S protein cleavage potentially and, consequently, post-protein transition. Therefore, the combined effect of these S protein mutations probably contributes toward increased viral fitness [16,21,22]. The N440K mutation has been observed in Clades 20A and 20B and has been found to cause re-infections, thus underscoring the importance of RBD mutations in enabling the virus to evade the immune system [23]. The accumulation of new mutations in the RBD possibly reflects the adaptive evolution of the virus in response to a growing proportion of the population with immunity to the Index virus [22]. The B.1.617.1 pangolin lineage (WHO label Kappa) with its characteristic spike mutation, L452R, was designated as a variant of interest (VOI) by the WHO on 4 April 2021 [24]. Thus, the surge in cases in the Amravati districts of Maharashtra could be associated with the presence of B.1.617.1 in the community.

The analysis of the temporal distribution of clades in Indian genomes (among the genomes submitted as of 28 February 2021) revealed the presence of B.1.617.1 as early as 31 January 2021. Following the detection of B.1.617.1 in the Amravati district, a lockdown was imposed on 22 February 2021 to contain the spread of this immune-evasive variant [25]. A study from Maharashtra indicated that the proportion of B.1.617.1 was 55-60% during February and March 2021. Simultaneously, the Kappa variant evolved into B.1.617.2, and the Delta variant comprised 10-60% of cases in Maharashtra and 3-10% in the rest of the country during the same period. Soon, the B.1.617.2 variant, driving the second wave, became the dominant variant in Maharashtra and India, spreading to other parts of the world [26]. The WHO designated the B.1.617.2 variant as a variant of concern (VOC) on 11 May 2021 due to its increased transmissibility, potential impact on vaccine efficacy, and increased severity of disease [27]. Following the initial lockdown in the Amravati district, public health interventions began on 28 March 2021 in Maharashtra, followed by a comprehensive lockdown in the state on 14 April 2021 [8]. These public health measures and the vaccination campaigns marked a critical phase in the battle against COVID-19 in India. However, the rapid emergence and the dominance of the Delta variant is a reminder of the virus’s adaptability and the need for continued surveillance and prompt public health response in effectively managing the pandemic.

## Conclusions

The COVID-19 pandemic has emphasized the critical role of genomic surveillance in understanding the spread and evolution of the virus. This study provides valuable insights into the circulating SARS-CoV-2 variants during the early second wave in Maharashtra, India. The distinct clustering of the samples from the Amravati district and the identification of the unique mutations of the Kappa variant enhance our understanding of the regional dynamics of the virus. Our findings emphasize the need for continuous monitoring and genomic surveillance to initiate timely public health measures to control the spread of the virus.

## Appendices

Appendix 1

Data Availability

GISAID identifier: EPI_SET_230918nw; doi: 10.55876/gis8.230918nw

All genome sequences and associated metadata in this dataset are published in GISAID’s EpiCoV database. To view the contributors of each individual sequence with details such as accession number, virus name, collection date, originating lab and submitting lab, and the list of authors, visit 10.55876/gis8.230918nw

Data Snapshot

EPI_SET_230918nw is composed of 1,474 individual genome sequences. The collection dates range from 2020-01-27 to 2021-01-11. Data were collected in one country and territories. All sequences in this dataset are compared relative to hCoV-19/Wuhan/WIV04/2019 (WIV04), the official reference sequence employed by GISAID (EPI_ISL_402124). Learn more at https://gisaid.org/WIV04.
